# 2,3,5,4′-Tetrahydroxystilbene-2-O-β-glucoside Attenuates Reactive Oxygen Species-Dependent Inflammation and Apoptosis in *Porphyromonas gingivalis*-Infected Brain Endothelial Cells

**DOI:** 10.3390/antiox11040740

**Published:** 2022-04-08

**Authors:** Vichuda Charoensaensuk, Yen-Chou Chen, Yun-Ho Lin, Keng-Liang Ou, Liang-Yo Yang, Dah-Yuu Lu

**Affiliations:** 1 School of Dentistry, College of Oral Medicine, Taipei Medical University, Taipei 11031, Taiwan; d825104004@tmu.edu.tw (V.C.); kevinyhl@tmu.edu.tw (Y.-H.L.); 2 Graduate Institute of Medical Sciences, College of Medicine, Taipei Medical University, Taipei 11031, Taiwan; yc3270@tmu.edu.tw; 3 3D Global Biotech Inc., New Taipei City 22175, Taiwan; klouyu@gmail.com; 4 Department of Physiology, School of Medicine, College of Medicine, China Medical University, Taichung 40402, Taiwan; 5 Laboratory for Neural Repair, China Medical University Hospital, Taichung 40447, Taiwan; 6 Department of Pharmacology, School of Medicine, College of Medicine, China Medical University, Taichung 40402, Taiwan; 7 Department of Photonics and Communication Engineering, Asia University, Taichung 41354, Taiwan

**Keywords:** 2,3,5,4′-Tetrahydroxystilbene-2-O-β-glucoside, *P. gingivalis*, ROS, inflammation, brain endothelial cells

## Abstract

We recently reported that the periodontopathic bacteria *Porphyromonas gingivalis* (*P. gingivalis*) initiates an inflammatory cascade that disrupts the balance of reactive oxygen species (ROS), resulting in apoptotic cell death in brain endothelial cells. An extract from *Polygonum multiflorum* Thunb., 2,3,5,4′-Tetrahydroxystilbene-2-O-β-glucoside (THSG) has been well-reported to diminish the inflammation in many disease models. However, the effects of THSG in the area of the brain–oral axis is unknown. In this study, we examined the effects of THSG in *P. gingivalis*-stimulated inflammatory response and apoptotic cell death in brain endothelial cells. THSG treatment remarkably lessened the upregulation of IL-1β and TNF-α proteins in bEnd.3 cells infected with *P. gingivalis*. Treatment of THSG further ameliorated brain endothelial cell death, including apoptosis caused by *P. gingivalis*. Moreover, the present study showed that the inhibitory effects on NF-κB p65 and antiapoptotic properties of THSG is through inhibiting the ROS pathway. Importantly, the ROS inhibitory potency of THSG is similar to a ROS scavenger *N*-Acetyl-L-Cysteine (NAC) and NADPH oxidase inhibitor apocynin. Furthermore, the protective effect of THSG from *P. gingivalis* infection was further confirmed in primary mouse brain endothelial cells. Taken together, this study indicates that THSG attenuates an ROS-dependent inflammatory response and cell apoptosis in *P. gingivalis*-infected brain endothelial cells. Our results also suggest that THSG could be a potential herbal medicine to prevent the risk of developing cerebrovascular diseases from infection of periodontal bacteria.

## 1. Introduction

Periodontitis is a major infection of the periodontium’s supporting components, which include the gingiva, cementum, alveolar bone, and periodontal ligament [1]. *Actinobacillus actinomycetemcomitans*, *Tannerellaforsythia*, *Prevotella*, *Fusobacterium*, and *Porphyromonas gingivalis* (*P. gingivalis*) are major Gram-negative etiological pathogens of periodontitis [1]. Among them, *P. gingivalis* is prominent for the progression of periodontitis [2]. Recently, the interconnection between oral microorganisms and brain disorders is an area of growing interest. Increasing epidemiologic reports have suggested a correlation between periodontal disease and systemic infection, particularly cardiovascular and cerebral ischemia diseases [3,4]. Previous findings reported that the pathogenesis of gingivitis and periodontitis increased the risks of cerebral ischemia diseases [5]. In the *P. gingivalis*-infected patients, elevated stroke risk has been observed [6]. Moreover, severe attachment loss, deeper periodontal pocket, an elevated score for plaque indices, significant bleeding, along with the increased levels of *P. gingivalis* were detected in patients with stroke, suggesting that this gum disease is associated with an increased risk of stroke [7]. During periodontitis, inflammatory cytokines, bacteria, and its virulence factors are released from the inflamed periodontium, thus promoting the innate immune response, endothelial impairment, and monocyte recruitment, thereby triggering the onset of stroke [4,8]. Periodontal disease and its pathogens may contribute to systemic diseases and neuroinflammation through several proposed mechanisms, including the transduction of systemic inflammation from oral inflammation, the interaction between host and microbial network, and bacteremia [9]. Bacteremia is a term referred to as the circulating bacteria in the bloodstream. Periodontal pathogens and their virulence factors can enter the systemic circulatory system via perturbed tissues and from daily routines (such as tooth brushing) and dental operation (such as scaling and tooth extraction) [10]. Our recent study provides evidence to support the link between the brain–oral axis, showing that *P. gingivalis* triggers an inflammatory response through the oxidative-stress pathway, thus causing apoptosis in brain endothelial cells [11]. 

An imbalance between free radicals, such as reactive oxygen species (ROS) and reactive nitrogen species (RNS), and antioxidant defenses is referred to as oxidative stress [12], which may cause a variety of cell deaths. Overproduction of ROS might lead to DNA and protein injury, inflammation, tissue damage, and cellular apoptosis [13], and also increase numerous pathologies in the brain that lead to various neurodegenerative diseases [14]. Clinical studies have proposed a strong correlation between ROS-induced oxidative stress and the Alzheimer’s disease (AD) pathogenesis [15]. Our recent finding also showed that ROS production in microglial cells is a critical factor in neuroinflammation [16]. A systemic increase in oxidative stress has been shown to relate to the progression of periodontal disease [17,18]. Moreover, the pathogenesis of gingivitis and periodontitis may be one of the important contributors to inflammatory conditions in the central nervous system (CNS) [19,20] and cerebrovascular diseases [21,22]. Moreover, several studies confirmed that periodontal treatment reduces circulating ROS and oxidative stress [23,24]. The NF-κB protein belongs to the transcription-factor family that triggers the expression of proinflammatory cytokines [25,26] and regulates immunological and inflammatory responses [27]. ROS activate NF-κB via IκBα phosphorylation with or without the degradation of IκBα. ROS also affect the DNA-binding properties of NF-κB protein [28]. Furthermore, the production of cytochrome c and proapoptotic proteins by mitochondrial-generated ROS triggers caspase activation, which leads to apoptosis [29].

*Polygonum multiflorum* Thunb. (Heshouwu) has long been recognized in traditional Chinese medicine as a tonic and antiaging agent. 2,3,5,4′-tetrahydroxystilbene-2-O-β-D-glucoside (THSG) is among the major components isolated from *Polygonum multiflorum* Thunb. [30]. THSG and resveratrol share a similar structure as members of the hydroxystilbene group, which exerts many pharmacological effects in cardiovascular and neurological systems [30,31]. Importantly, compared to resveratrol, THSG offers stronger antioxidant and free-radical-scavenging activities [30]. In addition, signal-transduction mechanisms engaged in the therapeutic actions of THSG include modulation of NF-κB [32,33] and suppression of intracellular ROS production [34]. Besides the ability to decrease ROS generation, THSG has also been reported to protect against cardiotoxicity by inhibiting apoptotic pathways [35]. Accumulating studies have reported that THSG possesses protective effects against neurological diseases [36,37], ischemia injury [32], atherosclerosis [38,39], and other diseases [40]. Administration of THSG has been observed to protect neuronal apoptosis in neuropathic pain [41] and Parkinson’s disease [42] mouse models. In the CNS, THSG has been found to inhibit inflammatory responses in brain microglial cells [43] and produce trophic factors in astrocytes [44]. Furthermore, pharmacological studies have suggested that THSG possesses numerous biological functions in aging-related CNS diseases, including cerebral ischemia, learning and memory disorders, and Alzheimer’s and Parkinson’s diseases [45].

A previous study also reported that THSG treatment increased cell viability of TNF-α-induced death of human umbilical-vein endothelial cells (HUVECs) [46]. We have recently reported that *P. gingivalis*-induced cell death in brain endothelial cells and periodontal infection may increase the risk of developing cerebrovascular diseases [11]. Importantly, previous studies also showed that THSG had a better efficiency in the prevention of periodontitis compared to resveratrol [34]. Currently, no study reports the effect of THSG on the brain–oral axis yet. This study aimed to investigate the effects of THSG treatment in *P. gingivalis* infection in brain vascular cells. The potential protective effect of THSG in *P. gingivalis*-stimulated inflammatory responses and cell apoptosis in brain endothelial cells were investigated. We further applied antioxidant agents including NAC and apocynin, which scavenge the production of ROS, to compare the antioxidative effect of THSG in cerebrovascular diseases.

## 2. Materials and Methods

### 2.1. Cell Culture

The ATCC^®^ CRL-2299™-immortalized mouse brain-derived endothelial cell line (bEnd. 3; ATCC; Manassas, VA, USA) was cultivated using DMEM basal medium (Dulbecco’s modified eagle medium; Cat. # 12100046; Gibco, Grand Island, NY, USA). The medium contained Penicillin-Streptomycin (Pen-Strep) antibiotic solution (1%; Cat. # 30-002-CI; Corning, Corning, NY, USA) and growth serum (FBS; 10%; Cat. # 26140079; Gibco, Grand Island, NY, USA). Cells were maintained in a controlled-atmosphere incubator (5% CO_2_, 95% air, 37 °C).

### 2.2. Primary Cell Isolation and Culture

All the animal-related procedures were handled in compliance with the Animal Care and Use Guidelines of China Medical University (Taichung, Taiwan). The animal protocols used in this study were issued by the Institutional Animal Care and Use Committee of China Medical University (CMUIACUC-2020-277). 

Seven to eight-week-old male C57BL/6 mice from BioLASCO (Taipei, Taiwan) were used to prepare primary mouse brain endothelial cells (MBECs) as described in our previous report [11]. Briefly, male C57BL/6 mice at 6-to-7 weeks old were obtained and caged in humidity- and temperature-regulated housing with ad libitum access to water and food for one week before the isolation. For each isolation, 15 mice were anesthetized and decapitated. Brains were transferred to a container filled with DMEM containing 2% Pen Strep. Prior to the mechanical digestion, cerebellum, olfactory bulb, and meninges were carefully removed. Brains were minced and homogenized using the 18-G needle followed by the 21-G needle. DNase type I (58.5 U/mL; Cat. # DN25; Sigma-Aldrich, St. Louis, MO, USA), collagenase type II (1.05 mg/mL; Cat. # C6885; Sigma-Aldrich, St. Louis, MO, USA), and DMEM were mixed and added to the homogenate for digestion. The homogenate was shaking at 200 rpm in a 37 °C incubator. The solution of 20% BSA (bovine serum albumin; Cat. # A9647; Sigma-Aldrich, St. Louis, MO, USA) was used to separate neuronal myelin sheath from the remaining parts by 1000× *g* centrifugation for 20 min at 4 °C. Pellets were collected and digested for a second time for 75 min in DMEM containing DNase type I (39 U/mL) and a mixture of collagenase/dispase (1 mg/mL; Cat. # 10269638001; Roche, Basel, Switzerland) on a 37 °C shaker incubator (200 rpm). Microvessels were separated from the contaminants by centrifuging the pellets (700× *g*, 10 min, 4 °C) in a continuous gradient of 33% Percoll (Cat. # 17089102; GE Healthcare, Chicago, IL, USA). Percoll residues were washed out. Then, microvessel fragments were plated onto collagen IV-coated plates (5 μg/cm^2^; Cat. # C5533; Sigma-Aldrich, St. Louis, MO, USA). The isolated brain endothelial cells were cultured in DMEM containing Pen-Strep antibiotic solution (1%), ITS supplement solution (Insulin-Transferrin-Sodium Selenium supplement; 0.2%; Cat. # I3146; Sigma-Aldrich, St. Louis, MO, USA), FBS (20%), sodium heparin (100 μg/mL; Cat. # H3393; Sigma-Aldrich, St. Louis, MO, USA), bFGF (basic fibroblast growth factor; 1 ng/mL; Cat. # ab217391; Abcam, Cambridge, UK), hydrocortisone (1.4 μM; Cat. # H0888; Sigma-Aldrich, St. Louis, MO, USA), and puromycin (4 μg/mL; Cat. # P8833; Sigma-Aldrich, St. Louis, MO, USA) in a controlled-atmosphere incubator (5% CO_2_, 95% air, 37 °C). After the first two days of culture, puromycin was removed from the culture medium. Once the cells reached 90–95% confluency, the dissociating recombinant enzyme TrypLE™ (Cat. # 12604021; Gibco, Grand Island, NY, USA) was used to subculture the cells.

The surface expression of CD31 (PECAM-1) was determined to confirm the purity of MBECs after the isolation and culture. Different passages of cells were harvested and stained with the FITC anti-mouse CD31 monoclonal antibody (Cat. # 102405; BioLegend, San Diego, CA, USA). Cells positively expressed CD31 were quantified by a flow cytometer (FACSCelesta™; BD, Franklin Lakes, NJ, USA). 

### 2.3. Bacterial Culture and Preparation

The *Porphyromonas gingivalis* ATCC^®^ 33277™ (*P. gingivalis*; ATCC; Manassas, VA, USA) was grown in TSB (tryptic soy broth; Cat. # 7164A; Acumedia, Lansing, MI, USA) and CDC anaerobe 5% sheep blood agar (Dr.plate, Neihu, Taipei, Taiwan) under an anaerobic condition in a 37 °C incubator. After cultivation, *P. gingivalis* was centrifuged, washed with phosphate buffer saline (PBS), and dispersed in serum- and antibiotic-free cell-culture medium. Bacteria were applied immediately to the cells for a live condition or treated with heat at 80 °C for 10 min [47] for a heat-killed bacteria condition. The number of bacteria added to the cells was calculated as the level of the multiplicity of infection (MOI).

### 2.4. 2,3,5,4′-Tetrahydroxystilbene-2-O-β-glucoside (THSG) Preparation

*Polygonum multiflorum* Thunb. was purchased from Chuang Song Zong pharmaceutical co. ltd (Kaohsiung, Taiwan) and identified by Industrial Technology Research Institute, Taiwan. The voucher specimens (He Shou Wu 01) of dried rhizoma were deposited at the Graduate Institute of Pharmacognosy, College of Pharmacy, Taipei Medical University, Taipei, Taiwan. The extraction and purification of THSG from *Polygonum multiflorum* Thunb was performed by Dr. Yu-Tang Chin and Dr. Ching-Chiung Wang [34].

### 2.5. Antioxidant Treatment and Bacterial Infection

Cells were pretreated with THSG in DMSO at a concentration of 0, 30, 100, or 200 µM for 2 h. To avoid the significant toxicity and side effects of DMSO on the cells, the final concentration of DMSO used in this study was lower than 0.1% *v*/*v*, which is well-tolerated with no observable toxic effects to endothelial cells [48,49,50]. In a separate experiment, cells were pre-incubated for 2 h with THSG (100 µM), NAC (*N*-Acetyl-L-cysteine; 10 mM; Cat. # A9165; Sigma-Aldrich, St. Louis, MO, USA), or apocynin (4′-Hydroxy-3′-methoxyacetophenone; 100 µM; Cat. # A10809; Sigma-Aldrich, St. Louis, MO, USA) in a plain-culture medium. After antioxidant treatment, heat-killed bacteria (MOI = 500) or live bacteria (MOI = 200) were added to the monolayer of cells. *P. gingivalis* was removed after 90 min. Cells were washed with PBS two times. Afterward, cells were cultivated in freshly added culture medium for the different time periods mentioned specifically in each experiment. 

### 2.6. MTT Assay

Cell viability following THSG and antioxidant treatment in *P. gingivalis*-infected cells was assessed by a Thiazolyl Blue Tetrazolium Bromide (MTT) assay. The protocol was modified from Ko et al. [51]. The bEnd.3 and MBECs (10,000 cells/mL) were plated onto a 24-well plate. THSG, NAC, or apocynin was added to the cells and incubated for 2 h, followed by 90 min of *P. gingivalis* infection. At the end of the infection period, *P. gingivalis* was discarded. Cells were washed twice with PBS and cultured in a fresh medium for 24 h. MTT (Thiazolyl Blue Tetrazolium Bromide; 5 mg/mL; Cat. # M5655; Sigma-Aldrich, St. Louis, MO, USA) was dissolved in PBS to prepare a working solution. Thereafter, MTT working solution (30 μL) and DMEM (270 μL) were mixed and placed in each well of the 24-well plate. Then, the plate was incubated for 1–2 h at 37 °C until water-insoluble formazan crystals formed. DMSO (Cat. # 802912; Merck Millipore, Billerica, MA, USA) was used to dissolve formazan crystals. The solutions were transferred to a 96-well plate. The absorbance at a wavelength of 540 nm was measured by Epoch Microplate Spectrophotometer (BioTek, Winooski, VT, USA). The number of viable cells after THSG treatment at different dosages was compared with the control (untreated) group and infection with THSG 0 µM group. The survival rate was calculated and is shown as the percentage of control.

### 2.7. Nuclear Staining

The bEnd.3 and MBECs ($5\times 10^{4}$ cells/mL in each well of a 6-well plate) were prepared one night before the experiment. Cells were treated with THSG for 2 h before *P. gingivalis* infection. After 24 h of incubation, paraformaldehyde in PBS (4%; Cat. # PB0684; Bio Basic, Toronto, ON, Canada) and Triton X-100 in PBS (0.1%; Cat. # AT1050-0500; Bionovas Biotechnology, Toronto, ON, Canada) were used to fix and permeabilize the cells, respectively (10 min each, at room temperature). Cell nuclei were probed with DAPI or 4′,6-diamidine-2′-phenylindole, dihydrochloride (1 μg/mL; Cat. # 71-03-01; SeraCare Life Sciences, Milford, MA, USA) for 10 min at room temperature. After two washes with PBS, the IX73 inverted system microscope (Olympus, Shinjuku, Tokyo, Japan) was used to visualize the nuclear condensation. Photos were obtained using VisiView imaging software version 09-2016 (Visitron Systems GmbH, Puchheim, Germany). 

### 2.8. FITC Annexin V and PI Staining

In addition to nuclear condensation, analysis of cell apoptosis was performed using a commercially available kit for apoptosis detection (Cat. # 556547; BD, Franklin Lakes, NJ, USA). The bEnd.3 and MBECs at $1\times 10^{6}$ cells/mL were cultured overnight in a 10-cm and 6-cm culture dish, respectively, before being treated with THSG, NAC, or apocynin for 2 h. *P. gingivalis* was allowed to infect cells for 90 min. Cells were washed twice with PBS and replaced with a new culture medium and incubated for another 24 h. Cells were harvested and resuspended in 1XAnnexin V binding buffer. One hundred microliters of the solutions were transferred to 5 mL round-bottom tubes. Then, 5 µL of FITC Annexin V and propidium iodide (PI) were added to the cells. Cells were incubated in the dark at room temperature for 15 min. A binding buffer (400 μL) was added and proceeded to the analysis with a flow cytometer (FACSCelesta™ or FACSCanto™ II; BD, Franklin Lakes, NJ, USA) with the following setup for compensation and quadrant: unstained cells, cells only stained with Annexin V conjugated with FITC, and cells only stained with PI. Phosphatidylserine (PS) is a membrane phospholipid located at the inner side of the cell membrane. During the early stage of apoptosis, the PS translocase enzyme triggers the relocation of PS to the outer side of the cell membrane, thus allowing Annexin V, which has a strong binding affinity for membrane phospholipid PS, to attach [52]. In addition, PI was used to exclude live cells from dead or damaged cells. In the latest stage of apoptosis, where membrane integrity is compromised, PI is permeable to the cells. In this study, cells with positively stained FITC Annexin V and negatively stained PI (quadrant 2; early apoptosis), and cells with positively stained FITC Annexin V and PI (quadrant 4; late apoptosis), were used to calculate the percentage of total apoptotic cells.

### 2.9. Western Blot Analysis

Proinflammatory cytokine proteins (from total cellular extracts) and NF-κB signaling pathway-related proteins (from nuclear/cytoplasmic and total cellular extracts) were analyzed using the Western blot technique. Briefly, bEnd.3 and MBECs ($1\times 10^{6}$ cells/mL) were added to a 10-cm or a 6-cm petri dish, respectively, and maintained for one night. Cells were rinsed with PBS for two times. A new medium was supplemented to the cells and cultured for an additional 15 min or 24 h after treatment and infection. At the end of an incubation period, cells were collected. A protease inhibitor cocktail (Cat. # P8340; Sigma-Aldrich, St. Louis, MO, USA) was added to a RIPA (radioimmunoprecipitation assay) lysis buffer (Cat. # 20-188; Merck Millipore, Billerica, MA, USA). Then, total cellular extracts were prepared by lysing cells for 1 h on ice with RIPA, and cell lysates were centrifuged at 4${\;}^{{}^{\circ}}C$ (20 min, 13,000× *g*). The supernatants were transferred to new microcentrifuge tubes for analysis. Extracts of nuclear/cytoplasmic proteins were prepared using a commercial extraction kit for nuclear protein (Cat. # ab113474; Abcam, Cambridge, UK) according to the manufacturer’s protocol. The concentration of total cellular protein and nuclear/cytoplasmic protein extracts were quantified by Pierce^TM^ BCA protein-assay kit (Cat. # 23225; Thermo Fisher Scientific, Waltham, MA USA). Protein samples were denatured at 95${\;}^{{}^{\circ}}C$ for 7 min with LaemmLi sample buffer (Cat. # 161-0747; Bio-Rad Laboratories, Hercules, CA, USA) containing 2-Mercaptoethanol (Cat. # 161-0710; Bio-Rad Laboratories, Hercules, CA, USA). Then, sodium dodecyl sulfate (SDS) polyacrylamide gel (8%, 10%, or 12%) was used to separate proteins according to their molecular weight. PVDF (polyvinylidene fluoride) membrane (Cat. # ISEQ00010; Merck Millipore, Billerica, MA, USA) was used to transfer proteins from the gel for antibody probing. The membranes containing proteins were incubated for 1 h with 3% BSA in TBST (Tris-buffered saline and Tween 20) at room temperature with agitation. TNF-α antibody (Cat. # ab66579; Abcam; Cambridge, UK); β-actin (Cat. # sc-47778) and IL-1β (Cat. # sc-7884) antibodies from Santa Cruz Biotechnology (Dallas, Texas, USA); PARP (Cat. # GTX100573), IκBα (Cat. # GTX110521), and NF-κB p65 (Cat. # GTX102090) antibodies from GeneTex (Irvine, CA, USA) were diluted at a suggested dilution factor and added to membranes overnight at 4${\;}^{{}^{\circ}}C$ with shaking. After the incubation with primary antibodies, goat anti-rabbit IgG H&L HRP (Cat. # ab6721; Abcam, Cambridge, UK) or goat anti-mouse IgG-HRP (Cat. # GTX213111-01; GeneTex, Irvine, CA, USA) was added to the membranes for 1 h at room temperature. The membranes were submerged with Immobilon Western Chemiluminescent HRP Substrate (Cat. # WBKLS0500; Merck Millipore, Billerica, MA, USA). Targeted protein bands were examined using ChemiDoc™ Imaging System (Bio-Rad Laboratories, Hercules, CA, USA) or BioSpectrum^®^ Imaging System™ (UVP, Upland, CA, USA). Protein band density was quantified by software from Image-Pro^®^ Plus (Media Cybernetics, Rockville, MD, USA).

### 2.10. NF-κB p65 Transcription Factor Assay

The activity of NF-κB p65 in nuclear extracts was further examined using the NF-κB p65 transcription-factor-assay kit (Cat. # ab133112; Abcam, Cambridge, UK). This method quantifies NF-κB activity based on the binding of NF-κB contained in nuclear extracts to the NF-κB-specific double-stranded DNA sequence precoated on the well plate. The assay was performed accordingly to manufacturer’s protocol. Briefly, the 96-well plate was firstly prepared by adding a binding buffer for the transcription factor to each well. Then, the nuclear lysates prepared previously for protein detection were pipetted to the designated wells. The plate was covered and left at 4 °C without agitation overnight. The solution was removed and rinsed with wash buffer 5 times. Primary antibody against NF-κB p65 transcription factor was added to the plate and incubated at room temperature without agitation for 1 h. The antibody solution was discarded. The plate was rinsed with wash buffer five times before being incubated for another hour at room temperature with a goat anti-rabbit secondary antibody against transcription factor conjugated with HRP. After incubation, the plate was rinsed with wash buffer five times. The HRP substrate was developed using a developing solution for the transcription factor. The plate was agitated with protection from light at room temperature during the process. The wells were allowed to turn medium to dark blue before adding a transcription-factor stop solution. The absorbance at 450 nm was read by Epoch Microplate Spectrophotometer. The DNA binding activity of NF-κB p65 was calculated in a fold of control and presented in a bar graph. 

### 2.11. Measurement of Reactive Oxygen Species (ROS) Generation

Intracellular production of ROS was detected by flow cytometer using DCFH-DA, as reported previously [51,53]. DCFH-DA is a cell-permeable nonfluorescent probe that converts to highly fluorescent DCF upon oxidation. Cells (bEnd.3 and MBECs) at $5\times 10^{5}$ cells/mL were plated on T25 flasks one night before treatment with THSG, NAC, or apocynin. Then, DCFH-DA (50 μM; Cat. # D6883; Sigma-Aldrich, St. Louis, MO, USA) was incubated with cells for 30 min. *P. gingivalis* was allowed to infect cells for another 90 min. After the infection, cells were collected enzymatically, dispersed in PBS, and analyzed with a flow cytometer (FACSCelesta™ or FACSCanto™ II; BD, Franklin Lakes, NJ, USA). 

### 2.12. Statistical Analysis

The experiments mentioned in this study were performed in quadruplicate. Values presented in the bar graphs are expressed as the mean ± standard error of the mean (SEM). Ordinary one-way analysis of variance (ANOVA) and Tukey’s multiple comparisons were used to analyze the significant differences between a study group. A *p*-value of less than 0.05 was defined as statistically significant. The statistical analysis software used in this study was from Graphpad Prism 9 for macOS (Graphpad Software, San Diego, CA, USA). 

## 3. Results

### 3.1. THSG Reduces Intracellular ROS Production and Increases Cell-Survival Rate in P. gingivalis-Infected Brain Endothelial Cells

**Figure 1:**
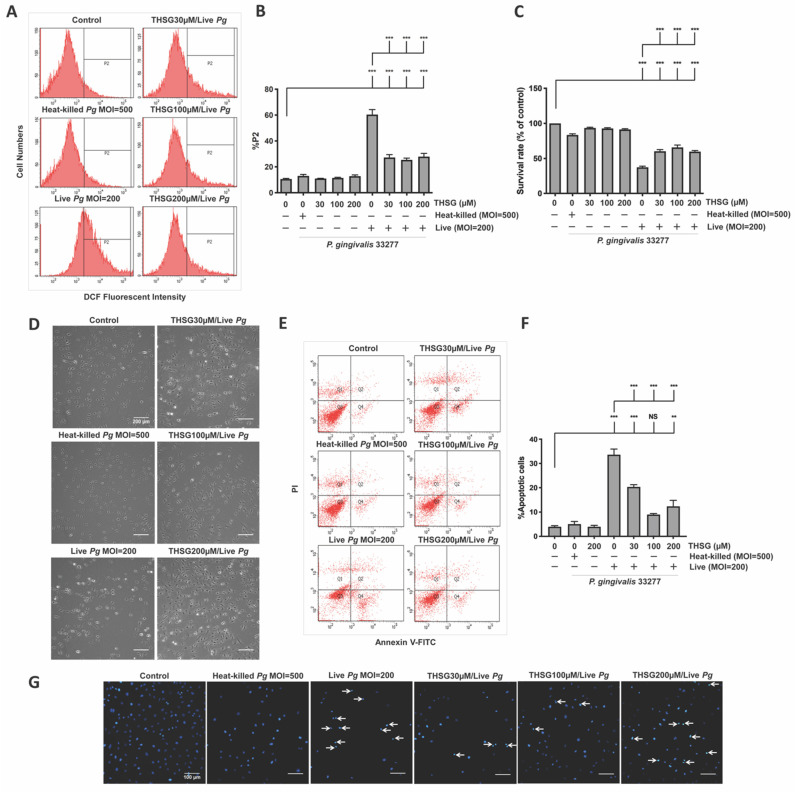
THSG attenuates *P. gingivalis*-triggered ROS production and cell death in bEnd.3 cells. THSG (0, 30, 100, and 200 µM) was used to pretreat the cells for 2 h before adding DCFH-DA (50 μM) for another 30 min. Heat-killed (MOI 500) or live (MOI 200) *P. gingivalis* were allowed to infect cells for 90 min. The DCF fluorescence intensity (P2 populations) representing ROS production was quantified using a flow cytometer (**A**,**B**). (**C**) The cell survival rate 24 h post-infection was examined by MTT assay, calculated, and expressed as a percentage of the control. (**D**) The morphology of cells was examined by a light microscope. Scale bar represents 200 μm. (**E**) Annexin V FITC/PI-stained cells were analyzed using a flow cytometer. The percentage of apoptotic cells is calculated and illustrated in (**F**). (**G**) Cells were stained with DAPI. Nuclear condensation was observed under a fluorescence microscope. Arrows are pointing at cells exhibiting condensed nuclei. Scale bar = 100 μm. Data are represented as means ± SEM (*n* = 4). Significant difference of the control and THSG 0 µM group are expressed as **, *p* < 0.01; *** and, *p* < 0.001. NS: not significant.

The percentage of the P2 population, which represented ROS-producing cells, was detected and shown in Figure 1A. After the infection with live bacteria, the %P2 was raised from 11% to 60% (Figure 1B). In addition, treatment with various concentrations of THSG significantly reduced *P. gingivalis*-induced ROS production to 27%, 25%, and 28%, respectively (Figure 1B). In addition, incubation with heat-killed bacteria did not affect ROS production (Figure 1A,B). We later examined whether THSG treatment protects brain endothelial cells from apoptotic cell death induced by *P. gingivalis*. The cell viability was quantified using an MTT assay (Figure 1C) and observed under a light microscope (Figure 1D). In the presence of live *P. gingivalis*, the survival rate was drastically decreased from 100% to 37% (Figure 1C). Moreover, treatment of various concentrations of THSG improved the survival rate of *P. gingivalis*-infected cells to 60%, 66%, and 60%, respectively. Moreover, we observed more intact cells following THSG treatment (Figure 1D). We later investigated whether THSG attenuates *P. gingivalis*-induced cell death by affecting cell apoptosis. Results of Annexin V FITC/PI staining showed a significant increase in the percentage of apoptotic cells caused by *P. gingivalis* in brain endothelial cells (Figure 1E), and cell apoptosis was reduced by THSG treatment (Figure 1E). The percentage of apoptotic cells in *P. gingivalis*-infected cells was elevated from 4% to 34% and lowered to 20%, 9%, and 10% following incubation with various concentrations of THSG (30, 100, 200 µM) (Figure 1F). Moreover, THSG at a concentration of 100 µM offered the best response as the percentage of apoptotic cells almost returned to that of a control group (*p* > 0.05 compared to the control group; Figure 1F). Furthermore, nuclear condensation as one marker of cell apoptosis was assessed by DAPI staining after *P. gingivalis* infection. The result showed that the occurrence of nuclear condensation in cells infected by *P. gingivalis* was clearly observed (Figure 1G); however, the phenomenon of nuclear condensation was markedly improved in cells treated with THSG. More healthy nuclei and fewer condensed nuclei were observed (Figure 1G). In addition, incubation with heat-killed bacteria did not affect Annexin V FITC/PI and DAPI staining (Figure 1F,G).

### 3.2. THSG Reduces the Upregulation of Proinflammatory Cytokines in P. gingivalis-Stimulated Brain Endothelial Cells

**Figure 2:**
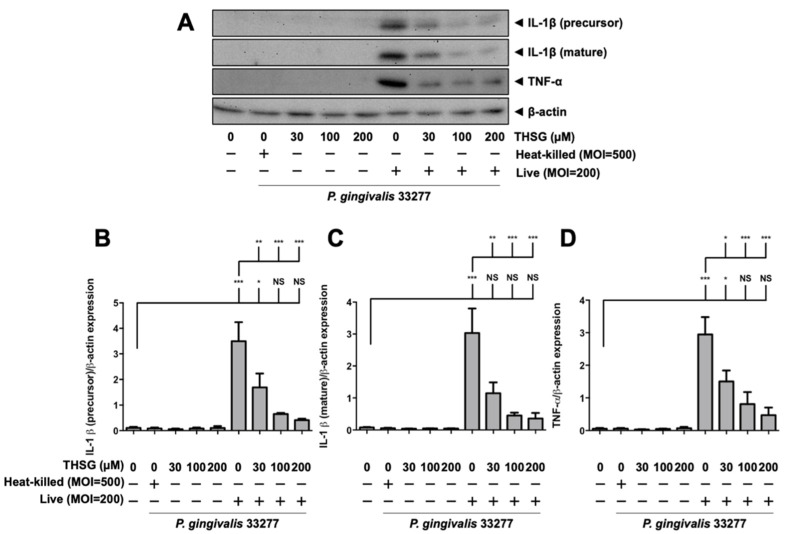
THSG reduces the expression of proinflammatory cytokines in *P. gingivalis*-infected bEnd.3 cells. (**A**) Cells were pretreated with THSG (0, 30, 100, and 200 µM) for 2 h before being challenged with heat-inactivated (MOI 500) or live (MOI 200) *P. gingivalis* for 90 min. The expression of IL-1β (precursor), IL-1β (mature), and TNF-α proteins 24 h after infection were examined using Western blot analysis. The quantitative results of IL-1β (precursor; (**B**)), IL-1β (mature; (**C**)), and TNF-α (**D**) expression were calculated from mean density per area and represented as bar graphs. Data are expressed as mean values ± SEM (n = 4). Significant difference of the control or THSG 0 µM group are presented as *, *p* < 0.05; **, *p* < 0.01; ***, and *p* < 0.001. NS: not significant.

THSG has been reported to offer anti-inflammatory properties in many cell types. In this study, we determined whether THSG improves *P. gingivalis*-upregulated IL-1β and TNF-α expression in brain endothelial cells or not. As shown in Figure 2, heat-killed *P. gingivalis* infection or THSG treatment alone did not affect expression of IL-1β (precursor), IL-1β (mature), and TNF-α proteins. However, upregulation of IL-1β (precursor), IL-1β (mature), and TNF-α were observed in *P. gingivalis*-infected brain endothelial cells. Furthermore, treatment with various concentrations of THSG (30, 100, 200 µM) significantly reduced the enhancement of IL-1β (precursor), IL-1β (mature), and TNF-α proteins in brain endothelial cells (Figure 2A–D). In addition, we observed a better anti-inflammatory effect of THSG at 100 and 200 µM as the expression of both cytokines returned to the normal state after treatment (*p* > 0.05 compared to the control group; Figure 2B–D). 

### 3.3. Treatment of THSG Inhibits NF-κB Signal Transduction Stimulated by P. gingivalis in Brain Endothelial Cells

**Figure 3:**
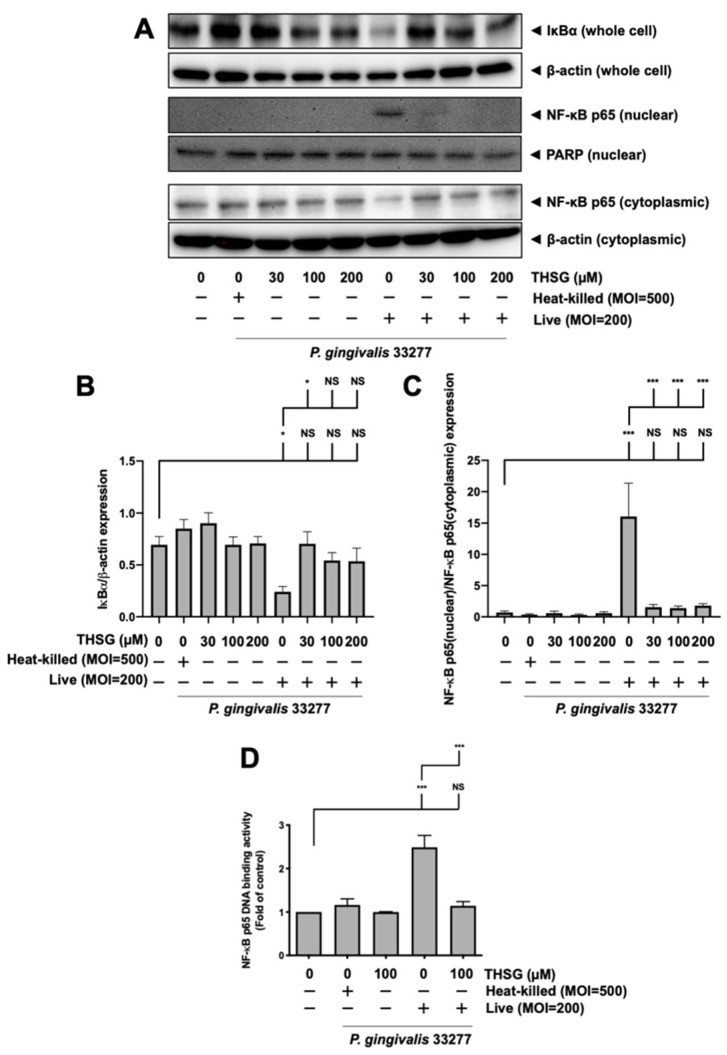
Suppression of NF-κB activation by THSG in *P. gingivalis*-infected bEnd.3 cells. THSG (0, 30, 100, or 200 µM) was added to cells 2 h before 90 min *P. gingivalis* infection (heat-killed MOI 500 or live MOI 200). Cell lysates were collected after 15 min of additional incubation with a fresh medium. (**A**) IκBα protein from whole-cell lysate and NF-κB p65 protein from nuclear and cytoplasmic fraction were analyzed by Western blot analysis. The quantitative results are shown in (**B**,**C**), respectively. (**D**) DNA binding activity of NF-κB p65 was determined by the transcription-factor assay and calculated in the fold of control. Data in bar graph are expressed as mean values ± SEM (n = 4). Significant difference of the control and THSG 0 µM group are presented as *, *p* < 0.05; ***, and *p* < 0.001. NS: not significant.

A transcription factor associated with inflammatory-response events, NF-κB, was further studied to examine signal transduction involved in the effect of THSG on *P. gingivalis*-stimulated inflammation and cell death in brain endothelial cells. The activation of NF-κB p65 was detected by Western blot analysis and the transcriptional activity of NF-κB was determined by DNA-binding activity assay. As shown in Figure 3A–C, *P. gingivalis* induced the degradation of IκBα protein expression and promoted NF-κB p65 protein translocation from cytoplasmic fractions to nuclear fractions. Moreover, THSG treatment dramatically restored the IκBα degradation (Figure 3B) and NF-κB p65 nuclear translocation (Figure 3C) in *P. gingivalis*-infected brain endothelial cells. Particularly, the expression levels of IκBα (Figure 3B) and NF-κB p65 (Figure 3C) proteins in cells with THSG treatment were not significantly different from cells in the control group.

Furthermore, *P. gingivalis* infection increased NF-κB p65 DNA-binding activity to approximately 2.5 fold (Figure 3D). The NF-κB transcriptional activity of cells infected with *P. gingivalis* in the presence of THSG treatment was significantly decreased (Figure 3D). In addition, the expression levels of IκBα, NF-κB p65 proteins, and NF-κB p65 DNA-binding activity were not affected by incubation of heat-killed bacteria.

### 3.4. The Reduction in ROS and NF-κB Activation Is Responsible for the Anti-Inflammatory and Antiapoptotic Properties of THSG in P. gingivalis-Infected Brain Endothelial Cells

**Figure 4:**
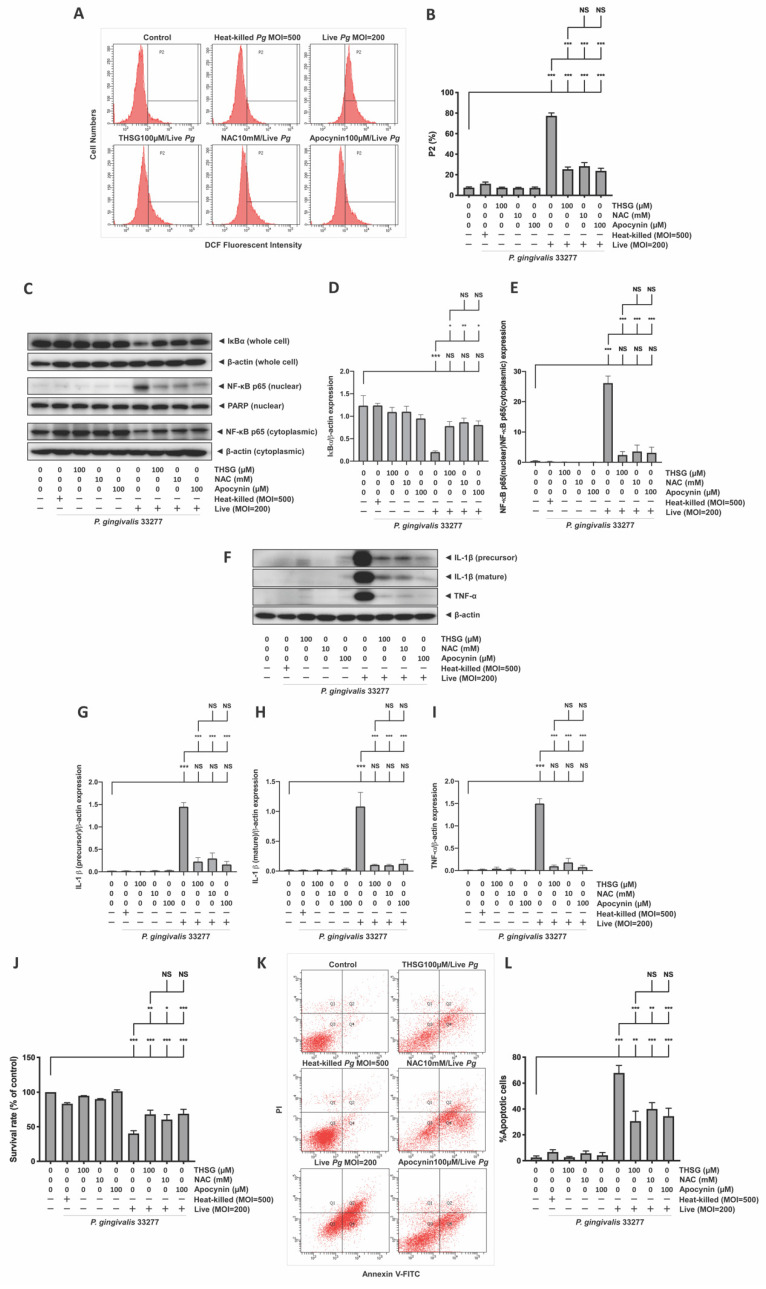
THSG treatment mitigates the toxicological effect of ROS production and NF-κB activation under *P. gingivalis* infection. bEnd.3 cells were treated with 100 µM THSG, 10 mM NAC, or 100 µM apocynin for 2 h before being challenged with *P. gingivalis* (MOI 200). (**A**) ROS production was elucidated by DCFH-DA (50 μM) and was analyzed using a flow cytometer. The quantitative results of DCF fluorescence intensity (P2 populations) are shown in (**B**). The protein expressions of IκBα and NF-κB p65 protein were analyzed by Western blot (**C**). The quantitative results of IκBα and NF-κB p65 are shown in (**D**,**E**). The protein expressions of IL-1β and TNF-α were detected by Western blot (**F**). The quantitative results of IL-1β and TNF-α are shown in (**G**–**I**). (**J**) The cell viability was examined by MTT assay in the percentage of control. (**K**) Cell apoptosis was determined by Annexin V FITC/PI staining and analyzed with a flow cytometer. The quantitative percentage of apoptotic cells are shown in (**L**). Data in bar graphs are expressed as mean values ± SEM (n = 4). Significant difference of the control, THSG 0 µM, THSG 100 µM group are presented as *, *p* < 0.05; **, *p* < 0.01; ***, and *p* < 0.001. NS: not significant.

Next, we further confirmed whether the anti-inflammatory and antiapoptotic effects of THSG are ROS-dependent. The effects of ROS scavenger *N*-Acetyl-L-cysteine (NAC) and NADPH inhibitor apocynin were used to compare with the protective effects of THSG. To determine the optimal concentration of apocynin for our study model, we treated brain endothelial cells with various concentrations of apocynin (50, 100, 200, and 300 µM). We did not observe cell toxicity when apocynin was administered alone at the indicated concentrations (Supplementary Figure S1A). The concentration of apocynin at 100 and 200 µM exhibited the best effects in the *P. gingivalis*-stimulated cell viability (Supplementary Figure S1A) and ROS production (Supplementary Figure S1B). Thus, we used apocynin at 100 µM in further studies. In addition, our previous study showed that NAC at 10 mM offered the best protective effects in brain endothelial cells [11]. The results in Figure 4A,B suggest that inoculation with live bacteria elevated %P2 to 77%, and THSG, NAC, and apocynin all antagonized the ROS generation caused by *P. gingivalis* in brain endothelial cells. Furthermore, treatment of THSG, NAC, and apocynin also prevented IκB degradation and NF-κB nuclear translocation in cells cultured with the bacteria infection (Figure 4C–E). In addition, the ROS production and NF-κB activity of cells incubated with heat-killed bacteria were similar to the control group. There are no significant differences in the protein levels of IκB and NF-κB in the groups of THSG, NAC, and apocynin treatment alone as well (Figure 4C–E). As a result, the expression of IL-1β and TNF-α proteins stimulated by the bacterial infection was improved following THSG, NAC, and apocynin treatment (Figure 4F–I). Accordingly, a significant increase in the survival rate (Figure 4J) and a significant decrease in the percentage of apoptotic cells (Figure 4K,L) were detected in *P. gingivalis*-infected cells treated with THSG, NAC, and apocynin. In addition, the survival rate of bacteria-infected cells increased from 40% to 68%, 60%, and 69% after the culture with THSG, NAC, and apocynin. The percentage of apoptotic cells in the bacteria infection was significantly decreased (Figure 4L). Treatment with THSG, NAC, and apocynin significantly improved the percentage of apoptotic cells in the bacteria-inoculated cells from 68% to 31%, 40%, and 35%, respectively (Figure 4L). Importantly, our results also showed that cotreatment of THSG and apocynin did not further improve the survival rate (Supplementary Figure S2A) and percentage of apoptotic cells (Supplementary Figure S2B). These results indicated that THSG shares the same effects as apocynin on *P. gingivalis*-induced ROS production. Furthermore, THSG and apocynin possess similar antioxidative potency. 

### 3.5. Anti-Inflammatory and Antiapoptotic Properties of THSG in P. gingivalis-Activated Primary Mouse Brain Endothelial Cells

**Figure 5:**
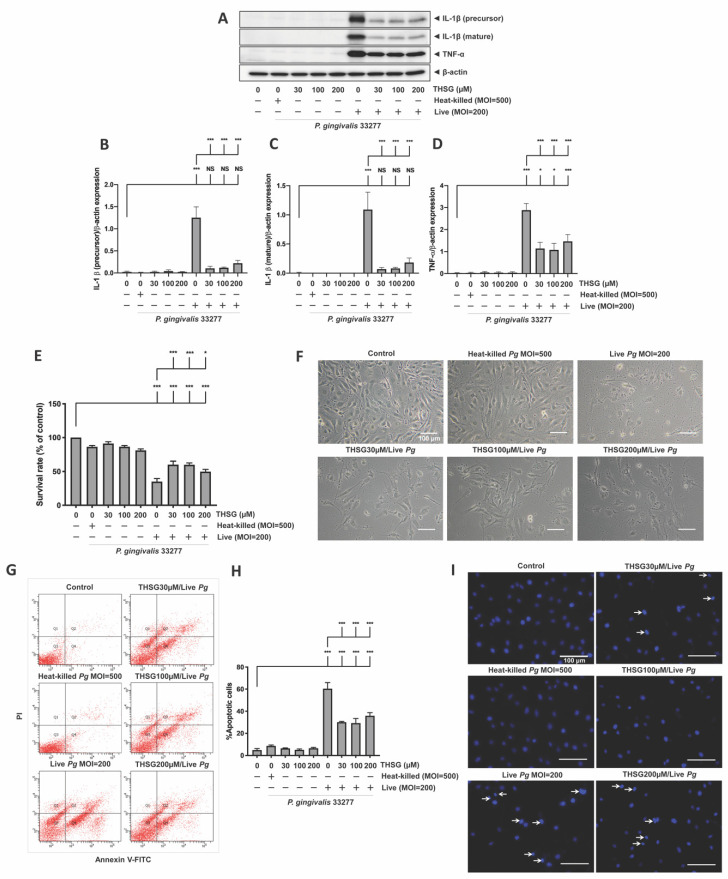
THSG possesses anti-inflammation and antiapoptosis in *P. gingivalis*-infected primary brain endothelial cells. MBECs were pre-incubated for 2 h with THSG (0, 30, 100, 200 µM) before 90 min *P. gingivalis* stimulation (heat-killed MOI 500, live MOI 200). After 24 h of additional incubation, the expression of IL-1β (precursor), IL-1β (mature), and TNF-α proteins were determined by Western blotting (**A**). The quantitative results are shown in (**B**–**D**). (**E**) The survival rate in a percentage of control was investigated using MTT assay. (**F**) Cell morphology was observed under a light microscope. MBECs were treated with THSG for 2 h and infected with *P. gingivalis*; apoptotic cells were determined by Annexin V/PI staining (**G**,**H**) and DAPI staining (**I**). All data are presented in mean value ± SEM (n = 4). *, *p* < 0.05; ***, *p* < 0.001; NS (not significant.), *p* > 0.05 were compared with control group and THSG 0 µM group. A scale bar is 100 μm in (**F**,**I**).

Next, to confirm the anti-inflammatory and anti-apoptotic properties of THSG, primary mouse brain endothelial cells (MBECs) were used in this study. As shown in Figure 5A–D, infection of *P. gingivalis* increased the expression of IL-1β (precursor), IL-1β (mature), and TNF-α proteins in MBECs. Moreover, treatment with various concentrations of THSG (30, 100, 200 µM) attenuated *P. gingivalis*-enhanced IL-1β precursor and mature forms (Figure 5A–C); and the expressions returned to the original levels. In addition, *P. gingivalis*-enhanced TNF-α protein expression was also decreased after THSG treatment (Figure 5A,D). Consequently, with THSG treatment, a drastic increase in cell-survival rate as measured by MTT assay (Figure 5E) and a light microscope (Figure 5F) was observed. Figure 5E showed that viable *P. gingivalis* (MOI 200) lessened the survival rate to 35%; THSG at 30, 100, and 200 µM was able to recover the survival rate back to 60%, 60%, and 50%, respectively. In addition, treatment with THSG alone did not cause a decrease in the cell viability as the survival rate remained similar to the control (Figure 5E). In Figure 5F, more viable cells were also observed in the bacteria-infected cells following THSG treatment. Moreover, experiments with annexin V and PI staining (Figure 5G,H) demonstrated that THSG (30, 100, and 200 µM) also dramatically reduced the percentage of apoptotic cells produced by *P. gingivalis* by approximately 50%. The percentage of apoptotic cells increased from 5% to 61% in the bacteria infection (Figure 5H). THSG at 30, 100, and 200 µM lowered the percentage of apoptotic cells in *P. gingivalis*-treated MBECs to 30%, 29%, and 36% (Figure 5H). Furthermore, the number of condensed nuclei after *P. gingivalis* infection also decreased in primary brain endothelial cells treated with THSG (Figure 5I). 

### 3.6. THSG Protects MBECs from Bacteria-Stimulated Inflammation and Apoptosis through ROS/NF-κB Signaling Pathway

**Figure 6:**
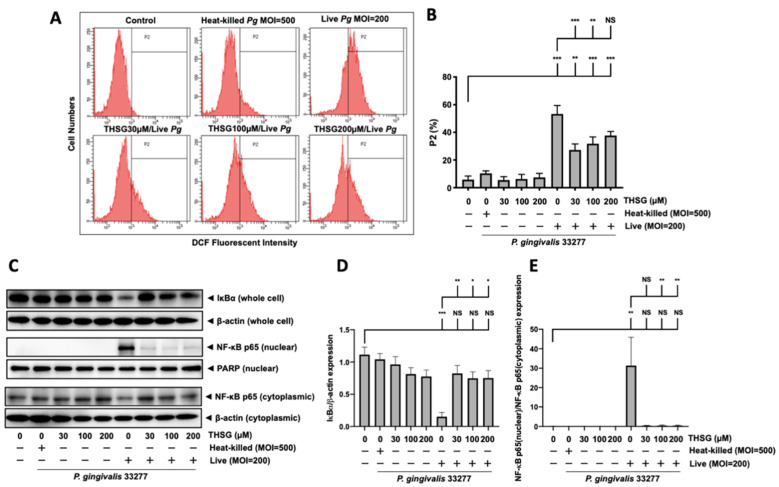
THSG reduces *P. gingivalis*-stimulated ROS production and prevents NF-κB activation in MBECs. (**A**) THSG (0, 30, 100, 200 µM) was incubated with cells for 2 h before adding DCFH-DA (50 μM) for another 30 min. Then, *P. gingivalis* (heat-killed MOI 500, live MOI 200) was introduced to cells, and the fluorescence intensity of DCF was calculated using a flow cytometer. The quantitative results of the gated histograms as the percentage of the P2 area are shown in (**B**). (**C**) The expression of IκBα from whole-cell lysates and NF-κB p65 from nuclear/cytoplasmic extracts of *P. gingivalis*-infected MBECs at 15 min after additional culture with fresh medium. The protein expressions were determined by Western blot, and the quantitative results are shown in (**D**,**E**). Data in the bar graph are expressed as mean values ± SEM (*n* = 4). Significant difference of the control and THSG 0 µM group are presented as *, *p* < 0.05; **, *p* < 0.01; ***, and *p* < 0.001. NS: not significant.

As shown in Figure 6A,B, the intracellular ROS levels of MBECs in *P. gingivalis* infection increased from 6% to 53%. Moreover, THSG treatment at 30, 100, and 200 µM reduced the ROS levels to 27%, 32%, and 38%, respectively. Moreover, we further observed that the degradation of IκBα protein due to bacterial infection was improved in MBECs with THSG treatment (Figure 6C,D). THSG treatment also reduced the enhancement of NF-κB p65 in nuclear proteins induced by *P. gingivalis* infection in MBECs (Figure 6C,E). 

## 4. Discussion

According to clinical and epidemiologic studies, periodontal disease has been considered a risk factor for several brain diseases [5,6,7]. Numerous studies have confirmed that alive and invasive periodontal pathogens can be detected in the brain and cause neuroinflammation and cognitive dysfunction. For instance, gingipains produced by *P. gingivalis* have been detected in the brains of patients with Alzheimer’s disease (AD) [54], and treatment of gingipain inhibitors may reduce the phenomenon [54]. In animal models, *P. gingivalis* inoculated into the palatal gingival tissues has been detected in the brain, which has induced Alzheimer’s disease-like pathology [55]. Moreover, *P. gingivalis* intravenously injected into rats causes neuroinflammation and Tau-protein hyperphosphorylation [56]. Surprisingly, *P. gingivalis* oral gavage-induced neuroinflammation and memory impairment in female C57BL/6J mice is age-dependent [57]. The present study used primary endothelial cells of C57BL/6 mice to examine the direct effect of a periodontal pathogen, *P. gingivalis*, on cell viability in vascular endothelial cells. Our results support periodontal disease and cerebrovascular inflammation based on the idea of bacteremia. 

There is growing evidence showing that cell apoptosis plays a significant role in periodontal disease and cerebrovascular disease. Cell apoptosis was observed in gingival epithelial [58] and fibroblast cells [59] that were infected with *P. gingivalis* and its virulence factors. *P. gingivalis* has been reported to cause cell apoptosis, which may possibly link a periodontal infection to vascular pathology [60]. Our recent study also found that *P. gingivalis* infection causes apoptotic cell death in brain endothelial cells, indicating that periodontal infection may increase the risk of developing cerebrovascular disease [11]. According to You et al. [61] and Zhong et al. [62], the extrinsic apoptosis pathway involves the NF-κB signaling pathway, caspase-8, -3, and Bid, while the intrinsic apoptosis pathway engages the caspase-9, -3, and BCL-2 family. The extract from *Polygonum multiflorum* was reported to improve the activation of caspase-8, caspase-3, and Bid in glutamate-treated primary cortical neurons, suggesting that the extract protects neurons through extrinsic pathway [63]. Moreover, THSG lowered the cleavage of caspase-3 in LPS-stimulated microglia [64]. In contrast, Lee et al. showed that THSG protected hippocampal neurons from glutamate-triggered toxicity through the intrinsic apoptosis pathway by regulating caspase-3 activation and the BCL-2 family [65]. *P. gingivalis* has been reported to cause cell apoptosis through both intrinsic and extrinsic apoptosis pathways via the modulation of caspase-9, -8, -3, Bax, and Bid [66]. Wang et al. [32] reported that THSG ranging from 10–100 µM significantly reversed the cytotoxicity effect and improved cortical apoptosis in the oxygen–glucose-deprivation and cerebral-ischemia models. Our study suggested that THSG attenuated *P. gingivalis*-induced apoptosis in brain endothelial cells. Moreover, the survival rate of *P. gingivalis*-infected brain endothelial cells with THSG pretreatment increased. The antiapoptotic activity of THSG in our model of the study was observed to be partial, yet there was a significant improvement in the outcome. 

Inflammation was devoted to the pathogenesis of stroke and periodontal inflammation [22,67]. *P. gingivalis* and its virulence factors were reported to upregulate inflammatory cytokines in several cells [34,68,69]. Increasing studies reported that THSG possesses anti-inflammatory effects in periodontal inflammation. Moreover, Chin et al. [34] reported a significant reduction in IL-1β, TNF-α, and IL-6 in *P. gingivalis* LPS-infected human gingival fibroblasts (HGFs) after treatment with THSG (1 and 25 µM) for 72 h. They also showed that THSG (0.1 and 10 mg/kg) significantly lowered the expression of IL-1β, TNF-α, iNOS, and COX-2 in ligature-induced experimental periodontitis in rats. Importantly, previous studies suggested that THSG reduces NF-kB activation that ameliorates the development of periodontitis [34]. In the present study, we found that treatment with THSG (30–200 µM) for 2 h attenuated *P. gingivalis*-induced NF-kB activation and expression of proinflammatory cytokines such as IL-1β and TNF-α proteins expression in brain endothelial cells. Our previous study supports this hypothesis by showing that a periodontal pathogen, *P. gingivalis*, causes inflammation and apoptotic death of brain endothelial cells through NF-κB/oxidative-stress pathway [11]. Accumulating studies have shown that a natural free-radical scavenger, 2,3,5,4′-Tetrahydroxystilbene-2-O-β-glucoside (THSG) isolated from *Polygonum multiflorum*, offers anti-inflammation, anti-atherosclerotic, and neuroprotective effects [70]. The present study supports previous studies by showing that THSG ameliorated *P. gingivalis*-induced inflammation and apoptosis in brain endothelial cells via the modulation of ROS and NF-κB activation. 

Overproduction of free radicals induces cell damage and eventually leads to many diseases such as atherosclerosis, stroke, cardiovascular disease, and cancer [13]. Recently, we have found that infection of *P. gingivalis* initiates ROS production and causes cell death in brain endothelial cells [11]. We previously also reported that an antioxidant, NAC, effectively scavenges ROS to reduce NF-κB activation and promote cell survival [11]. Apocynin is a natural polyphenolic compound with multiple biological activities, such as inhibition of NADPH oxidase [71]. Previously, it has been reported by several preclinical studies that apocynin offers a therapeutic effect in inflammatory-related diseases without any observed toxicity [72,73]. Oral treatment of apocynin has been reported to protect many neurodegenerative diseases [74]. Moreover, blood–brain-barrier damage following the middle cerebral-artery-occlusion model in rats was improved in rats treated with apocynin [75]. Interestingly, apocynin has been considered a complementary treatment for mild coronavirus disease 2019 (COVID-19) infection [76]. Apocynin mediates learning and memory deficit in Parkinson’s disease by inhibiting NADPH oxidase and NF-κB activation [77]. THSG has been reported to exhibit a strong free-radical-scavenging activity in the 2,2-diphenyl-1-picrylhydrazyl (DPPH) test [78]. Our study presented that upregulation of inflammatory cytokines and cell death started with the production of ROS during the infection with *P. gingivalis*, which was restored by treatment with THSG. 

The well-established free-radical scavenger NAC and the NADPH inhibitor apocynin were used to compare the ROS inhibitory effects of THSG on brain endothelial cells. THSG shares the general antioxidant and antiapoptotic mechanisms with NAC and apocynin by scavenging ROS that contribute to inflammation and cell death. Furthermore, THSG exerted similar effects compared to NAC and apocynin in preventing NF-κB activation and proinflammatory cytokine expression induced by *P. gingivalis* infection. Accordingly, a significant decrease in cell death and reduction in the percentage of apoptotic cells were observed in *P. gingivalis*-infected brain endothelial cells treated with NAC, apocynin, and THSG. However, the specific mechanisms underlying the effect of THSG and the synergistic effects of THSG and NAC or apocynin should be further investigated in the future study. Importantly, this study proved that the therapeutic effect of THSG is comparable to NAC and apocynin. The potency of THSG to reduce ROS production caused by *P. gingivalis* in brain endothelial cells is similar to NAC and apocynin. In addition, THSG at the dosages ranging from 1 to 200 µM was used to treat several types of the cell, including HGFs [34], rat cortical neurons [32], and HUVECs [46,79] without any observed toxicity. In the present study, brain endothelial cells were cultured with THSG at concentrations of 30 to 200 µM for 2 h. Our results suggested that THSG did not cause any toxicity to both types of brain endothelial cells used in this study. 

## 5. Conclusions

The present study showed the inhibitory effects of an extract of Chinese herbal material *Polygonum multiflorum*, THSG on the *P. gingivalis*-stimulated inflammatory response and apoptotic cell death in brain endothelial cells. THSG ameliorated *P. gingivalis*-activated expression of IL-1β and TNF-α and NF-κB activation in both bEnd.3 and primary brain endothelial cells. The protective effects of THSG in brain endothelial cells were through the reduction in ROS activation induced by *P. gingivalis.* The ROS inhibitory potency of THSG was compared with the well-known ROS scavenger NAC and ROS inhibitor apocynin. Taken together, THSG could be a potential herbal medicine to prevent the risk of developing a cerebrovascular disease caused by periodontal pathogen infection.

## Data Availability

Data is contained within the article and supplementary material.

## Supplementary Materials

The following supporting information can be downloaded at: https://www.mdpi.com/article/10.3390/antiox11040740/s1, Figure S1: Apocynin optimum dosage determination in brain endothelial cells. Figure S2: Effect of THSG and apocynin co-treatment on *P. gingivalis*-stimulated cell death in brain endothelial cells.
