# Supplementary material for: 2,3,5,4′-Tetrahydroxystilbene-2-O-β-glucoside Attenuates Reactive Oxygen Species-Dependent Inflammation and Apoptosis in Porphyromonas gingivalis-Infected Brain Endothelial Cells
Source: Antioxidants (Basel). 2022 Apr 8;11(4):740. doi: 10.3390/antiox11040740 (PMC9024880; doi:10.3390/antiox11040740)
Supplement: Supplementary file 1 [file antioxidants-11-00740-s001.zip › antioxidants-1634420-supplementary.pdf]

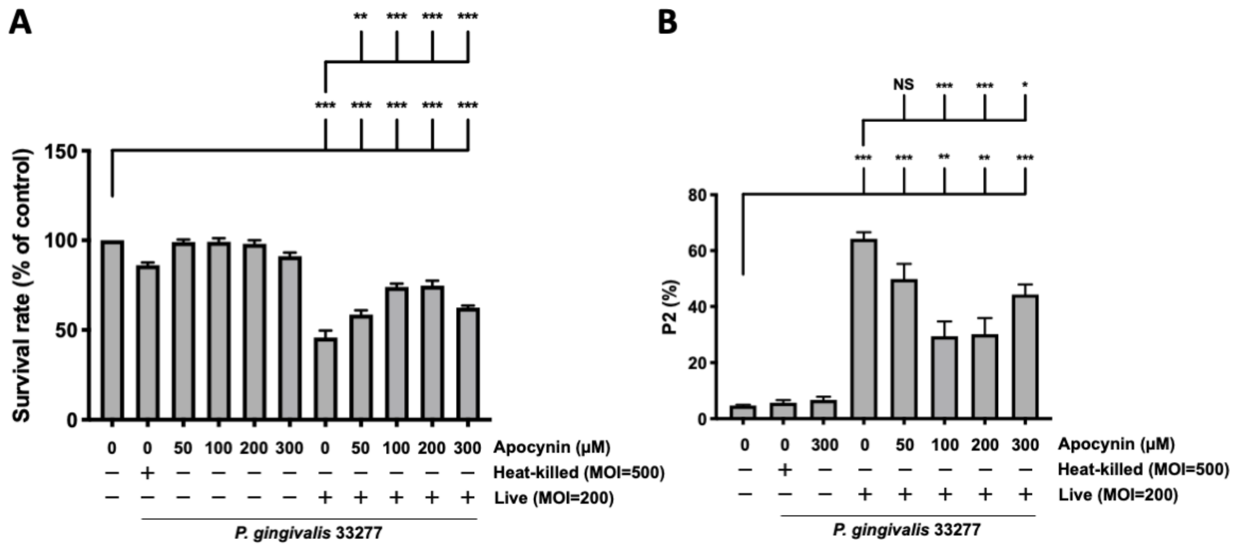

Supplementary Figure S1. Apocynin optimum dosage determination in brain endothelial cells. bEnd.3 cells were cultured with apocynin at 0, 50, 100, 200, and 300 μM before inoculating with *P. gingivalis* (heat-killed MOI 500 or live MOI 200). (**A**) The survival rate at 24 h post-infection was determined by MTT assay. (**B**) DCFH-DA (50 μM) was added to the cells before the infection to examine the intracellular production of ROS. The fluorescence intensity of DCF was calculated using a flow cytometer and presented as a gated histogram in the percentage of the P2 area. Data in the bar graph are shown as mean values ± SEM (n = 4). Significant difference of the control and apocynin 0 μM group are presented as *, $p < 0.05$; **, $p < 0.01$; ***, and $p < 0.001$. NS: not significant.

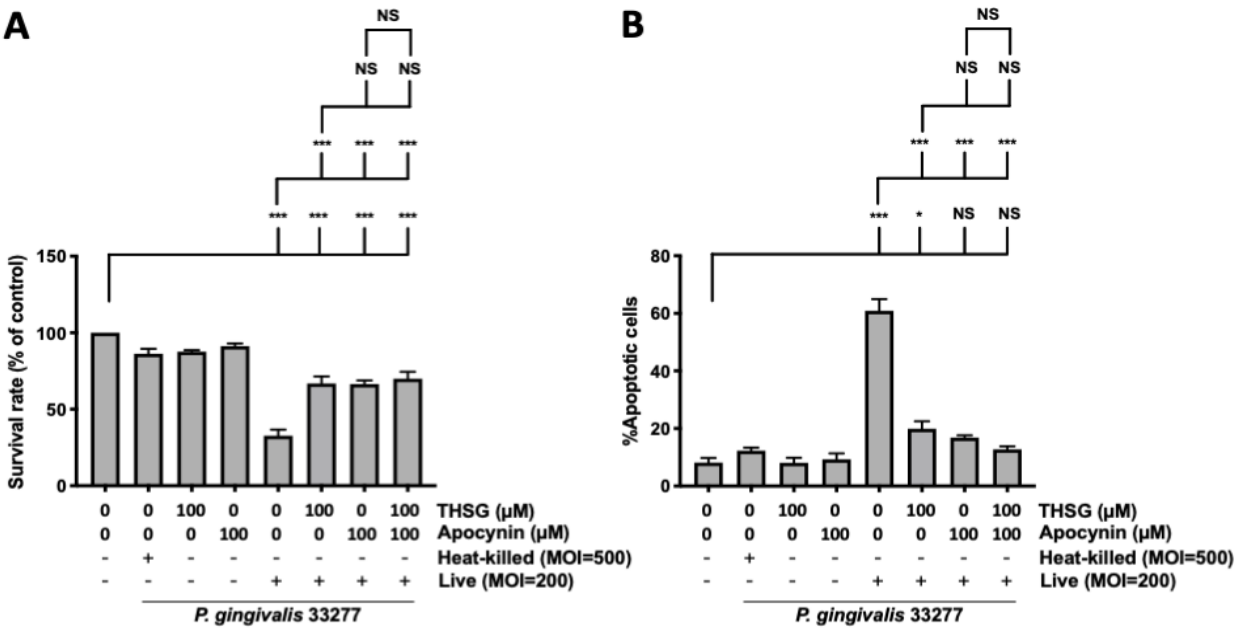

Supplementary Figure S2. Effect of THSG and apocynin co-treatment on *P. gingivalis*-stimulated cell death in brain endothelial cells. bEnd.3 cells were treated for 2 h with 100 μM THSG, 100 μM apocynin, or the combination of THSG and apocynin before being infected with heat-killed (MOI 500) or live (MOI 200) *P. gingivalis* for another 90 minutes. (**A**) Twenty-four hours after the infection, the survival rate was quantified by MTT assay and presented as a percentage of the control. (**B**) Annexin V FITC/PI was used to stain the cells to determine the percentage of apoptotic cells. Cells were analyzed using a flow cytometer. Data in bar graphs are represented as means ± SEM ($n = 4$). Significant difference of the control, infection with THSG 0 μM, infection with THSG 100 μM, and infection with apocynin 100 μM group are expressed as *, $p < 0.05$; ***, and $p < 0.001$. NS: not significant .
